# Inhibition of pro-apoptotic UPR pathways PERK/CHOP and IRE1/JNK protects differentiated SH-SY5Y cells against rotenone-induced toxicity

**DOI:** 10.3389/fnmol.2025.1700897

**Published:** 2025-12-03

**Authors:** Natalia Siwecka, Wioletta Rozpȩdek-Kamińska, Michał Golberg, Wojciech Wiese, Grzegorz Galita, Ireneusz Majsterek

**Affiliations:** 1 Department of Clinical Chemistry and Biochemistry, Medical University of Lodz, Lodz, Poland; 2 Department of Neurology, Medical University of Lodz, Lodz, Poland; 3 Department of Histology and Embryology, Medical University of Lodz, Lodz, Poland; 4 Department of Child Psychiatry, Medical University of Warsaw, Warsaw, Poland

**Keywords:** Parkinson’s disease, rotenone, ER stress, unfolded protein response, PERK, IRE1, JNK

## Abstract

**Introduction:**

Parkinson’s disease (PD) is a chronic neurodegenerative disorder characterized by loss of dopaminergic neurons and α-synuclein aggregation in the midbrain. One proposed mechanism in PD pathogenesis is endoplasmic reticulum (ER) stress followed by activation of the unfolded protein response (UPR). The UPR consists of three main branches, among which the protein kinase RNA-like ER kinase (PERK) and inositol-requiring enzyme 1 (IRE1) contribute to pro-apoptotic signaling by inducing C/EBP homologous protein (CHOP) and c-Jun N-terminal kinase (JNK), respectively.

**Methods:**

This study investigates the neuroprotective potential of selective inhibition of PERK/CHOP and IRE1/JNK signaling against rotenone (ROT)-induced toxicity in differentiated SH-SY5Y cells, an in vitro model of PD. For this purpose, the inhibitors of mentioned UPR pathways AMG44 and JNK V were applied, and their biological effect was examined in terms of cell viability, morphology, cell death, oxidative stress level, gene and protein expression profiles.

**Results:**

Exposure to ROT significantly decreased cell viability, disrupted cell morphology, induced reactive oxygen species generation, apoptosis, necrosis, and affected the expression of UPR-related factors, indicative of ER stress, oxidative damage and cell death. Treatment with AMG44 and JNK V significantly prevented or reversed these changes, and the underlying mechanism involved altered expression of the specific ER stress-related markers. Moreover, inhibition of one of the UPR pathways influenced the other, highlighting the crosstalk between PERK/CHOP and IRE1/JNK branches in ROT-induced neurotoxicity.

**Conclusion:**

Targeting PERK- and IRE1-dependent pathways contributes to neuroprotection in ROT-based PD model, which indicates the potential of UPR inhibitors as therapeutic agents for PD.

## 1 Introduction

Parkinson’s disease (PD) is a progressive, chronic neurodegenerative disorder that typically occurs later in life. Its prevalence increases with age, peaking in individuals aged ≥ 65 years (108–201 cases per 100,000) (Dorsey et al., 2018; Willis et al., 2022). PD pathology begins with neuronal degeneration and the accumulation of misfolded α-synuclein proteins inside nerve cells, forming Lewy bodies. Such aggregates are detected in 90% of clinically confirmed, post-mortem PD cases (Halliday, 2005). The disease affects specific regions of both central and peripheral nervous systems. In particular, neuronal loss occurs in the substantia nigra, an area rich in dopamine-producing brainstem neurons (Dickson et al., 2009; Tanner and Ostrem, 2024). The resulting dopamine depletion causes symptoms such as asymmetric bradykinesia with rigidity, resting tremor, and imbalance. The cause of PD still remains unknown. About 20% of PD cases have a described genetic component, while the other 80% are idiopathic (Tanner and Ostrem, 2024). Dysregulation in protein folding, trafficking, or metabolism is one suspected cause. This leads to endoplasmic reticulum (ER) luminal overload, called ER stress (Funayama et al., 2023; Ren et al., 2021). The ER proteostasis is vital for cell survival. Its disruption activates an adaptive mechanism called the unfolded protein response (UPR). The UPR pathway includes three ER membrane proteins: protein kinase RNA-like endoplasmic reticulum kinase (PERK), inositol-requiring enzyme 1 (IRE1), and activating transcription factor 6 (ATF6) (Chen et al., 2023). Under ER stress, these proteins direct the cell toward either pro-survival or pro-apoptotic arms of the UPR pathway. In PD, ATF6 mainly acts as a cytoprotective axis of the UPR. PERK and IRE1 mainly contribute to the degradation of dopaminergic neurons in PD (Credle et al., 2015; Egawa et al., 2011; Hashida et al., 2012; Kovaleva and Saarma, 2021; Puspita et al., 2017).

PERK and IRE1 dimerize and autophosphorylate in response to ER stress (Kopp et al., 2019). Phosphorylated PERK (p-PERK) acts on eukaryotic translation initiation factor 2α (eIF2α), inhibiting global protein translation and allowing selective translation of activating transcription factor 4 (ATF4) (Bond et al., 2020). ATF4 promotes expression of genes that mediate apoptosis, such as *DNA damage-inducible transcript 3 (DDIT3)*, which encodes C/EBP homologous protein (CHOP), and B-cell lymphoma 2 (Bcl-2) family proteins. CHOP promotes apoptosis by downregulating the anti-apoptotic factor Bcl-2 or by increasing reactive oxygen species (ROS) production (McCullough et al., 2001). The cytosolic domain of IRE1 acts as an endoribonuclease, splicing X-box binding protein 1 (XBP1) mRNA to generate XBP1s. XBP1s then activates expression of pro-survival genes (Kim et al., 2011). If ER stress is prolonged, IRE1 can trigger apoptosis through interaction with TNF receptor-associated factor 2 (TRAF2), activating apoptosis signal-regulating kinase 1 (ASK1) and c-Jun N-terminal kinase (JNK), both of which mediate apoptosis (Zeng et al., 2015). Activated JNK modulates Bcl-2 family members, including anti-apoptotic proteins such as Bcl-2 and Bcl-xL, as well as pro-apoptotic proteins like Bax and Bak (Yang and Yao, 2015).

To model PD features in vitro, experiments use Rotenone (ROT), a pesticide that causes neurotoxicity by inhibiting mitochondrial complex I (Pereira et al., 2023). ROT interferes with the mitochondrial electron transport chain. Specifically, it inhibits electron transfer from iron-sulfur centers in complex-I to ubiquinone and affects ATP synthesis (Choi et al., 2010; Goswami et al., 2016). Rotenone’s neurotoxicity comes from its ability to promote oxidative stress by generating ROS. This redox imbalance disrupts proteostasis, leads to abnormal protein folding, and induces ER stress (Goswami et al., 2016). Several reports show ER stress, UPR pathway activation, and JNK3 isoform involvement as central mechanisms in ROT-induced neural cell toxicity (Choi et al., 2010; Choi et al., 2015; Goswami et al., 2016; Ren et al., 2021; Ryu et al., 2002).

In this study, we investigated whether pharmacological suppression of PERK/CHOP and IRE1/JNK signaling modulates ROT-induced neurotoxicity in vitro. To gain insight into molecular pathways contributing to dopaminergic cell death, we used differentiated SH-SY5Y cells treated with ROT, along with the specific UPR inhibitors AMG PERK 44 and JNK V. Understanding these regulatory mechanisms in PD pathogenesis is fundamental and may lead to development of novel treatments for PD.

## 2 Materials and methods

### 2.1 Cell culture and differentiation

The study was performed on the human neuroblastoma SH-SY5Y cell line. The cell line present in this study was obtained from the American Type Culture Collection (ATCC; Catalog No. CRL-2266). This lineage represents a widely used in vitro model for PD and can be differentiated to achieve dopaminergic phenotype. The culture was maintained in DMEM:F-12 medium (1:1) containing 10% fetal bovine serum (ATCC) and 1% penicillin-streptomycin (ScienCell) in a humidified cell culture incubator (37°C, 5% CO_2_). The medium change was performed every 2–3 days and the cells were subcultured every 7 days (at ∼80% confluency) via detachment with 0.25% Trypsin/EDTA solution (ScienCell), centrifugation, and seeding at 1:5 ratio. The cell line did not reach the 15th passage, and in each experiment cells at the same passage number were used. The cells were differentiated in poly-L-lysine coated vessels by incubation with 10 μM retinoic acid (RA) under low-serum conditions for 7 days prior to experiments, as described previously (Siwecka et al., 2024).

### 2.2 Drug treatment

ROT was applied as the neurotoxic agent in all experiments. The study utilized two potent, highly selective inhibitors of the pro-apoptotic UPR-related kinases: Amgen compound 44 (AMG44) as a PERK inhibitor, and JNK V (AS601245) as an inhibitor of JNK, especially JNK3 isoform related to the central nervous system (CNS). All compounds were acquired from Sigma-Aldrich and their specifications are listed in Table 1. The compounds were reconstituted in DMSO (BioShop), and the stock solutions were kept at-20°C in the dark. In each experiment, ROT was used for 48 h, and the inhibitors were added for 1 h prior to or after ROT incubation. The concentrations of the compounds were chosen based on the cytotoxicity experiments results.

**TABLE 1:** The specification of the applied chemical compounds obtained from Sigma-Aldrich.

Compound name	Full name	Product no.	Structure
Rotenone	(2R,6aS,12aS)-8,9-Dimethoxy-2-(prop-1-en-2-yl)-1,2,12,12a-tetrahydro[1]benzopyrano[3,4-b]furo[2,3-h][1]benzopyran-6 (6aH)-one	R8875	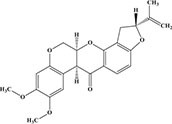
AMG PERK 44	4-(2-Amino-4-methyl-3-(2-methylquinolin-6-yl) benzoyl)-1-methyl-2,5-diphenyl-1H-pyrazol-3 (2H)-one hydrochloride, 4-[2-Amino-4-methyl-3-(2-methyl-6-quinolinyl) benzoyl]-1,2-dihydro-1-methyl-2,5-diphenyl-3H-pyrazol-3-one hydrochloride	SML3049	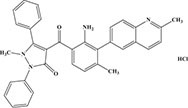
JNK Inhibitor V	1,3-Benzothiazol-2-yl-(2-((2-(3-pyridinyl) ethyl)amino)-4-pyrimidinyl)acetonitrile	420129	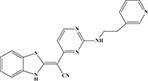

### 2.3 Cytotoxicity analysis

The effect of ROT and selected UPR inhibitors on viability of differentiated SH-SY5Y cels was assessed by two separate methods, namely XTT and LDH colorimetric assays. In the XTT assay (Invitrogen), the XTT tetrazolium sodium salt is conversed into orange-colored formazan product by living cells, which corresponds to the intensified absorbance value at 450/630 nm. Pierce LDH Cytotoxicity Assay Kit (Thermo Scientific) is based on the assessment of extracellular LDH released from damaged cells into the culture medium. The LDH level is quantified by a couple of enzymatic reactions, which involves the conversion of lactate to pyruvate by LDH with reduction of NAD^+^ to NADH, and subsequent reduction of tetrazolium salt (INT) to a red-colored formazan product by diaphorase and NADH; this results in increased absorbance at 490/680 nm. The cytotoxic effect of ROT was determined after 24 and 48 h incubation. The potential cytotoxicity of the tested inhibitors, AMG44 and JNK V, and of the compounds solvent (0.1% DMSO) was also determined in cells after 24 and 48 h incubation. In the next step, to assess whether PERK/CHOP and IRE1/JNK inhibition exerts protective effect upon ROT exposure, the inhibitors were applied as a pre-treatment (1 h) or post-treatment (1 h) to ROT-treated cells (48 h). After incubation with the compounds, the respective cytotoxicity assays were performed in accordance with the manufacturers’ protocols. The absorbance was measured at appropriate wavelengths by means of the Synergy HT Microplate Reader (BioTek). As part of the cytotoxicity analysis, the IC50 values for ROT and the inhibitors after 48 h of incubation were calculated by nonlinear regression of the dose–response relationship using Statistica 13.3 software (StatSoft, Tulsa, OK, United States).

### 2.4 Morphology assessment

The images of cell morphology were acquired using an inverted microscope (Nikon) at 10× magnification. The morphological changes were evaluated in untreated differentiated SH-SY5Y cells, cells treated with ROT (12.5 μM, 48 h), and cells exposed to ROT (12.5 μM, 48 h) and UPR inhibitors as a pre-and post-treatment (1 h) at the most effective concentrations, according to the cytotoxicity experiment results (pre-treatment: AMG44 at 0.4 μM, JNK V at 1.6 μM; post-treatment: AMG44 at 0.8 μM, JNK V at 0.8 μM). The quantitative evaluation of morphological changes was performed by measuring average neurite length (ANL) and cell attachment count under an inverted microscope using NIS-Elements Advanced Research software, version 5.42 (Nikon). The ANL value was calculated based on the measurement of the neurite length of 100 randomly selected cells. Cell attachment was quantified by counting the number of adherent cells per defined area using threshold-based segmentation followed by particle analysis. All image analyses for morphological assessment were performed by a qualified researcher blinded to experimental conditions to ensure unbiased quantification.

### 2.5 Evaluation of caspase-3 processing

The assess whether inhibition of PERK/CHOP and IRE1/JNK affects ROT-induced apoptosis in differentiated SH-SY5Y cells, the Caspase-3 colorimetric assay was applied (Abcam). Caspase-3 is regarded as an executioner caspase in cell apoptosis, as well as it is involved in ROT-induced neural cell death (Ahmadi et al., 2003). The assay is based on the quantification of p-nitroaniline (p-NA), a chromophore which is formed via cleavage from the labeled substrate DEVD-pNA. The Caspase-3 activity is then measured based on the absorbance of p-NA at 400 nm. The cells were treated with the compounds as described above. Untreated cells were used as a negative control, and cells treated with staurosporine (10 μM, 24 h)—as a positive control. Following the incubation, the cell lysis and Caspase-3 reaction were performed as described in the manufacturers’ protocol. The protein concentration was adjusted to 100 μg protein/sample using Pierce BCA Protein Assay (Thermo Scientific). The absorbance measurement was performed by the Synergy HT Microplate Reader (BioTek).

### 2.6 Evaluation of apoptosis

To further determine the effect of UPR inhibitors on the specific types of ROT-induced cell death in differentiated SH-SY5Y cells, the fluorescein (FITC)-conjugated Annexin V (AV)/propidium iodide (PI) fluorescent staining was applied (BD Pharmingen). The assessment is based on the affinity of AV to preferentially bind phosphatidylserine, which is exposed at the outer leaflet of membrane of apoptotic cells. On the other hand, PI passes through damaged membrane and enters nuclei to intercalate into nucleic acids in late apoptotic and necrotic cells. Therefore, the application of AV/PI counterstaining allows to distinguish viable (AV-/PI-), early apoptotic (AV+/PI-), late apoptotic (AV+/PI+) and necrotic (AV-/PI+) cells based on differences in plasma membrane integrity and permeability. After staining, AV+ apoptotic cells show green fluorescence, PI+ dead cells show red fluorescence, while live cells show little to no fluorescence (Koç et al., 2018). The cells were treated with the compounds as described above. Following the exposure, the cells were stained with FITC-AV, PI (BD Pharmingen) and Hoechst (Invitrogen) in the provided binding buffer for 15 min, according to the manufacturers’ protocol, and then the samples were analyzed under fluorescent microscope at 20 × magnification (Nikon). The image analysis for fluorescence quantification was performed by a qualified researcher who was blinded to the experimental conditions and treatment groups to ensure unbiased data evaluation.

### 2.7 Measurement of reactive oxygen species

In order to evaluate the effect of PERK and JNK inhibition on the level of ROT-induced reactive oxygen species (ROS), the fluorometric Reactive Oxygen Species (ROS) Detection Assay Kit (Abcam) was carried out. The assay detects hydroxyl, peroxyl and other ROS activity in cell using a fluorogenic, cell-permeable reagent 2’,7’-dichlorofluorescein diacetate (DCFDA), which is deacetylated by esterases upon cellular uptake and then oxidized by ROS into a highly fluorescent dye 2’,7’-dichlorofluorescin (DCF). Drug treatments were performed as described above; differentiated SH-SY5Y cells cultured in the complete medium only constituted a negative control, and cells incubated with ROS inducer 50 μM antimycin A (24 h) constituted a positive control. The assay was carried out as described in the manufacturer’s instruction; briefly, the cells were stained with ROS Label at 37°C for 45 min in the dark prior to drug treatment. Following the incubation, the fluorescence intensity was measured at Ex/Em = 485/535 nm using the Synergy HT spectrophotometer (BioTek).

### 2.8 Gene expression analysis

The changes in expression of certain genes associated with PERK- and IRE1-dependent signaling were determined by the qRT-PCR analysis in differentiated SH-SY5Y cells treated with the selected UPR inhibitors and ROT. The treatments with the compounds were carried out as described above, and the control cells were left untreated. Following the drug treatment, the total RNA was extracted from cells using PureLink RNA Mini Kit (Invitrogen) as described in the manufacturer’s protocol and quantified using the Synergy HT Microplate Reader (BioTek). The isolated RNA was then transcribed into cDNA (100 ng) by the High-Capacity cDNA Reverse Transcription Kit (Applied Biosystems) according to the manufacturer’s guidelines. The expression profiles of the following genes: *DDIT3*, *XBP1*, *MAPK10*, *EIF2A*, *BCL2*, and *GAPDH* were analyzed by the respective TaqMan Gene Expression Assays (Applied Biosystems), as listed in Table 2. The reaction mix (20 μL) consisted of cDNA (1 μL), TaqMan probes (1 μL), TaqMan Universal PCR Master Mix II (10 μL) (Applied Biosystems), and nuclease-free water (8 μL) (Invitrogen), and the PCR reaction was run by the Bio-Rad CFX96 detection system (Bio-Rad) involving the step of initial denaturation (95°C, 15 min), followed by 40 cycles of cycling—denaturation (95°C, 10 s), and 40 cycles of annealing/extension (60 °C, 60 s). The data was quantified using 2^–ΔΔCt^ method.

**TABLE 2:** The ID numbers of the applied TaqMan™ Gene Expression Assays (Applied Biosystems).

Gene name	Encoded protein	Assay ID
*DDIT3*	CHOP	(Hs01090850_m1)
*XBP1*	XBP1	(Hs00231936_m1)
*MAPK10*	JNK3	(Hs00959268_m1)
*EIF2A*	eIF2α	(Hs00230684_m1)
*BCL2*	BCL2	(Hs00608023_m1)
*GAPDH*	GAPDH	(Hs99999905_m1)

### 2.9 Protein expression analysis

Immunoblotting was performed to assess the effect of PERK/CHOP and IRE1/JNK inhibition on the expression levels of the selected ER stress-related factors upon ROT exposure. Differentiated SH-SY5Y cells were exposed to ROT and UPR inhibitors as described previously, apart from control cells that were left without any treatment. The total protein was isolated from cells by Minute™ Total Protein Extraction Kit (Invent Biotechnologies), and the normalization of protein amount was performed using Pierce™ BCA Protein Assay (Thermo Scientific). Then the protein samples were denatured (70 °C, 10 min), underwent electrophoresis and subsequent transfer onto PVDF membrane utilizing the NuPage™/XCell SureLock™ system (Invitrogen). Following the transfer, the membranes were blocked for 1 h in 5% BSA (for phosphoproteins) or skim milk (for non-phosphoproteins) in 1X TBST (Thermo Scientific Chemicals). Overnight, the membranes were incubated at 4°C in BSA/milk 1X TBST solution with primary monoclonal antibodies against p-eIF2α (1:500), eIF2α (1:1,000), p-JNK (1:500), JNK (1:1,000), XBP1s (1:200), CHOP (1:1,000), p-Bcl-2 (1:500), Bcl-2 (1:1,000), β-actin (1:1,000). Next, the membranes were rinsed three times with 1X TBST and incubated with the secondary HRP-linked antibodies (1:5,000) at RT for 1 h. All antibodies were acquired from Cell Signaling Technology and are listed in Table 3. The enhanced chemiluminescence (ECL) method was applied to visualize the immune complexes, which were detected using ChemiDoc Imaging System (Bio-Rad). The protein bands were quantified using densitometry in NIS-Elements Advanced Research software (Nikon).

**TABLE 3:** The catalog numbers of the applied antibodies obtained from the Cell Signaling Technology.

Antibody name	Dilution	Catalog No.
Phospho-eIF2α (Ser51) antibody	1:500	#9721
eIF2α (D7D3) rabbit mAb	1:1,000	#5324
CHOP (D46F1) rabbit mAb	1:1,000	#5554
XBP-1s (D2C1F) rabbit mAb	1:200	#12782
SAPK/JNK antibody	1:1,000	#9252
Phospho-SAPK/JNK (Thr183/Tyr185) antibody	1:500	#9251
Bcl-2 (D55G8) rabbit mAb	1:1,000	#4223
Phospho-Bcl-2 (Ser70) (5H2) rabbit mAb	1:500	#2827
β-Actin (13E5) rabbit mAb	1:1,000	#4970
Anti-rabbit IgG, HRP-linked antibody	1:5,000	#7074

### 2.10 Data analysis and statistics

Data analysis was performed in Statistica 13.3 software (StatSoft). The data distribution was determined by Shapiro–Wilk test, whereas the homogeneity of variance—by Levene’s test. In all experiments except for ANL measurement, the obtained data were normally distributed and homogenous, and therefore parametric statistical tests were applied to determine statistical significance, specifically one- or two-way ANOVA with Bonferroni correction. For ANL quantification, the Kruskal–Wallis test with *post-hoc* Dunn’s test was performed. All experiments were conducted in triplicate. In the graphs, the values are presented as mean ± SD (or median and interquartile range for ANL), and the significance is displayed as *p < 0.05, **p < 0.01, and ***p < 0.001.

## 3 Results

### 3.1 Evaluation of the compounds’ cytotoxicity

**FIGURE 1:**
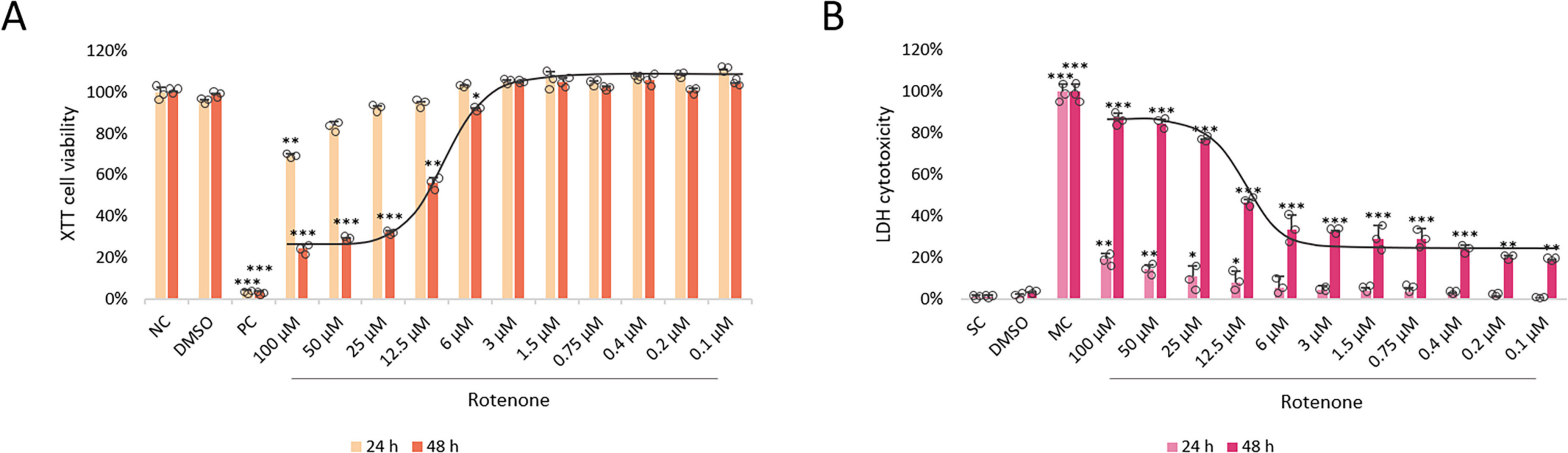
The XTT cell viability **(A)** and LDH cytotoxicity **(B)** analyses of the neurotoxin rotenone (ROT) in differentiated SH-SY5Y cells. Cells were treated with ROT at concentrations 100–0.1 μM for 24 and 48 h, and then XTT test (A) and LDH test (B) were performed. The corresponding dose-response curves are presented for 48 h ROT treatment, IC50 = 14.2 μM **(A)**, IC50 = 13.6 μM **(B)**. Data were analyzed by the one-way ANOVA with Bonferroni correction. Values are presented as mean ± SD (*n* = 3), *p < 0.05, **p < 0.01, ***p < 0.001 vs. negative control **(A)** or spontaneous control **(B)**. NC, Negative Control, Untreated Cells; DMSO, Solvent Control, cells treated with 0.1% Dimethyl Sulfoxide; PC, Positive Control, cells treated with 20% dimethyl sulfoxide; SC, Spontaneous LDH Activity Control, cells treated with water; MC, Maximum LDH Activity Control, cells treated with 10X Lysis Buffer.

The cytotoxicity of ROT in differentiated SH-SY5Y cells was assessed by two methods, namely XTT assay and LDH assay. For this purpose, the cells were incubated with the neurotoxin for 24 and 48 h in wide concentration range (100–0.1 μM), which was determined based on the previous literature data. Additionally, the ROT solvent (0.1% DMSO) was also tested for toxicity, and it did not significantly affect cell viability as assessed by the two assays. XTT assay revealed significant induction of cytotoxicity by ROT at 100 μM after 24 h, and at the concentrations ≥ 6 μM after 48 h. In LDH assay, ROT induced significant damage at ≥ 12.5 μM concentrations after 24 h incubation and all concentrations after 48 h. In all cases the cytotoxic effect of ROT was dose-dependent. The differences between the two tests could be explained by higher sensitivity of LDH assay to detecting low-level cell membrane damage, which is one of the known mechanisms of ROT-induced toxicity (Sun et al., 2021). As we were interested in long-term toxicity induced by ROT, and due to the fact that at 48 h ROT induced significantly more damage, the 48 h incubation time was chosen for further experiments. The 12.5 μM concentration of ROT was chosen for further testing as it induced significant amount of damage (∼50%) as detected by both assays. The exact IC50 values for the 48 h ROT treatment have also been calculated, amounting to 14.2 μM in the XTT assay and 13.6 μM in the LDH assay (Figure 1).

**FIGURE 2:**
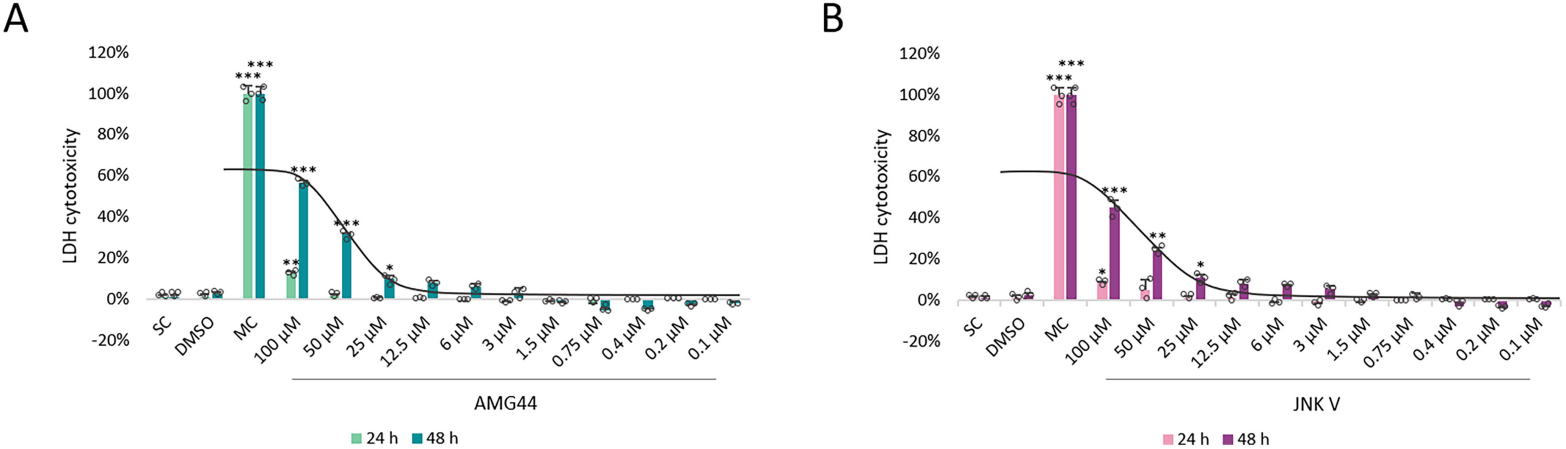
The cytotoxicity analysis of PERK inhibitor AMG44 **(A)** and JNK inhibitor JNK V **(B)** in differentiated SH-SY5Y cells by LDH assay. Cells were treated with AMG44 or JNK V at concentrations 100–0.1 μM for 24 and 48 h, and then LDH test was performed. The corresponding dose-response curves are presented for 48 h treatment with the inhibitors, IC50 = 80.5 μM for AMG44 **(A)**, IC50 = 122.2 μM for JNK V **(B)**. Data were analyzed by the one-way ANOVA with Bonferroni correction. Values are presented as mean ± SD (*n* = 3), *p < 0.05, **p < 0.01, ***p < 0.001 vs. spontaneous control. SC, Spontaneous LDH Activity Control, cells treated with water; DMSO, Solvent Control, cells treated with 0.1% Dimethyl Sulfoxide; MC, Maximum LDH Activity Control, cells treated with 10X Lysis Buffer.

In accordance with the literature data and our previous experiments, we evaluated the potential cytotoxicity of the AMG44 and JNK V inhibitors in differentiated SH-SY5Y cells at concentration range of 100–0.1 μM and at 24 and 48 h incubation time. We previously performed XTT cell viability analysis for the tested compounds in differentiated SH-SY5Y cells, which demonstrated inhibition of cell proliferation at relatively high concentrations ( = 50 μM) and induction of cell proliferation by the inhibitors at lower concentrations, indicating a hormetic effect (Siwecka et al., 2024). Similarly, LDH test showed induction of cytotoxicity at = 25 μM at 48 h incubation and a slight reduction in extracellular lactate level induced by lower concentrations of both compounds, although insignificant. Therefore, the non-toxic low concentrations of the inhibitors ( = 12.5 μM) were chosen for further testing. The IC50 values for both compounds at 48 h of incubation were 80.5 μM for AMG44 and 122.2 μM for JNK V (Figure 2).

### 3.2 UPR inhibitors increase the viability of ROT-treated cells

**FIGURE 3:**
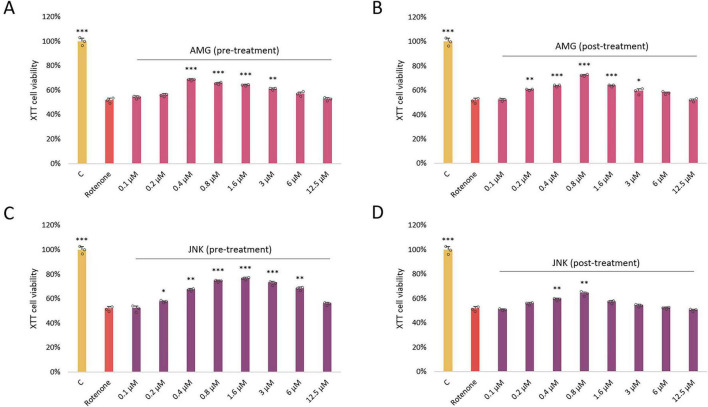
The effect of AMG44 pre-treatment **(A)** or post-treatment **(B)** and JNK V pre-treatment **(C)** or post-treatment **(D)** (1 h each) on the viability of differentiated SH-SY5Y cells incubated with the neurotoxin rotenone (ROT) at 12.5 μM for 48 h. The inhibitory compounds were tested at 12.5–0.1 μM concentration range. Cell viability was evaluated by XTT assay. Data were analyzed by the one-way ANOVA with Bonferroni correction. Values are presented as mean ± SD (*n* = 3), *p < 0.05, **p < 0.01, ***p < 0.001 vs. ROT. C, Control, untreated cells; AMG, Cells Treated with ROT (48 h) and AMG44 inhibitor (1 h); JNK, Cells Treated with ROT (48 h) and JNK V inhibitor (1 h).

**FIGURE 4:**
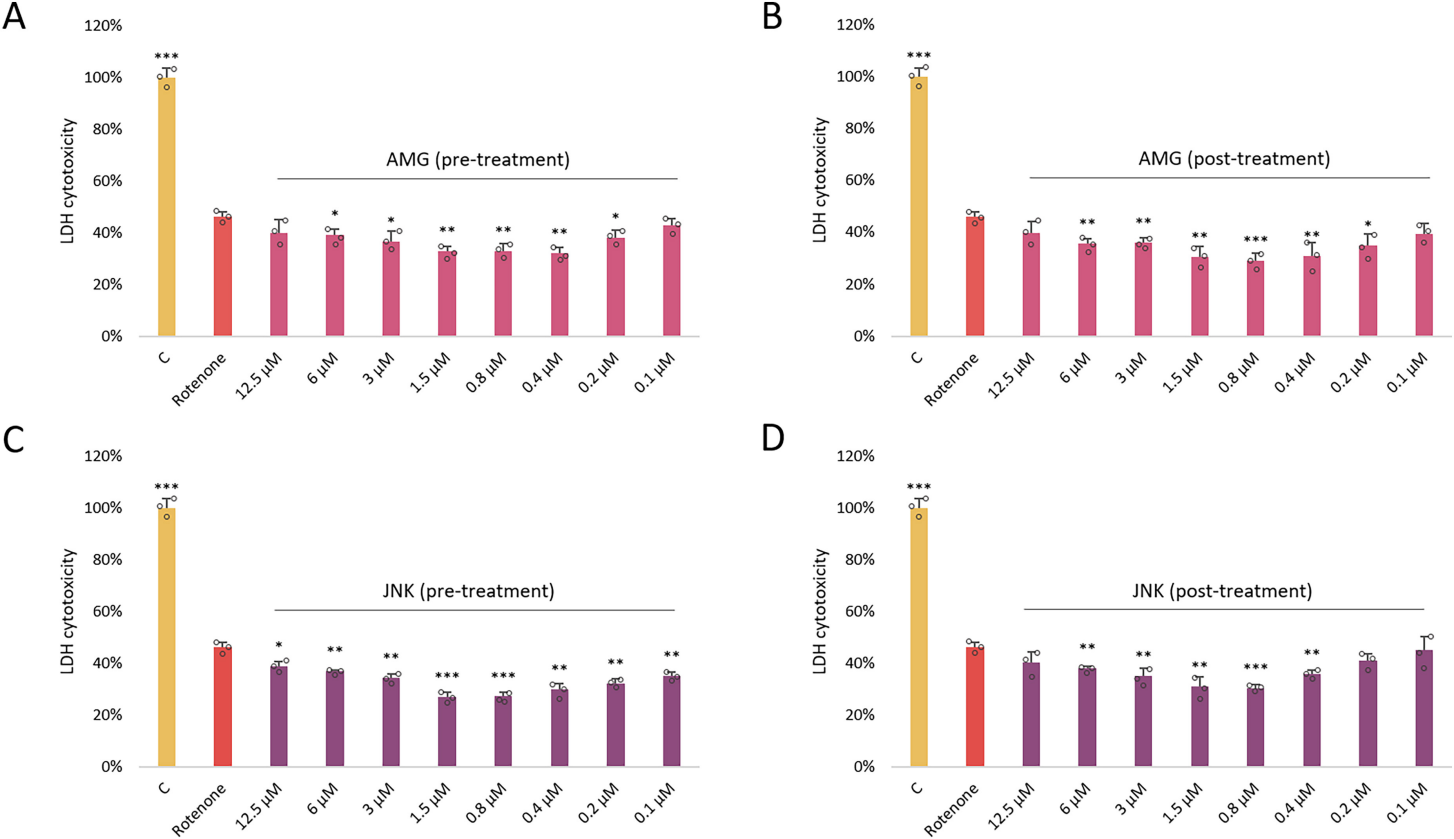
The effect of AMG44 pre-treatment **(A)** or post-treatment **(B)** and JNK V pre-treatment **(C)** or post-treatment **(D)** (1 h each) on cytotoxicity in differentiated SH-SY5Y cells incubated with the neurotoxin rotenone (ROT) at 12.5 μM for 48 h. The inhibitory compounds were tested at 12.5–0.1 μM concentration range. The cytotoxicity was evaluated by LDH assay. Data were analyzed by the one-way ANOVA with Bonferroni correction. Values are presented as mean ± SD (*n* = 3), *p < 0.05, **p < 0.01, ***p < 0.001 vs. ROT. C, Control, cells treated with 10X Lysis Buffer; AMG, cells treated with ROT (48 h) and AMG44 inhibitor (1 h); JNK, cells treated with ROT (48 h) and JNK V inhibitor (1 h).

Once we knew the effect of each drug on cell viability, we aimed to assess whether inhibition of PERK/CHOP or IRE1/JNK axis of the UPR affects ROT-induced toxicity. As in the previous experiment, the two separate cytotoxicity assays XTT and LDH were applied. Differentiated SH-SY5Y cells were exposed to ROT at 12.5 μM for 48 h and incubated with the inhibitory compounds AMG44 and JNK V at the 12.5–0.1 μM range for 1 h before or after ROT-induced damage. Each of the compounds significantly protected cells against ROT toxicity at certain concentrations when they were used as a pre-treatment, and significantly reversed ROT-induced injury when applied as a post-treatment. This indicated that both PERK/CHOP and IRE1/JNK pathways were implicated in ROT-induced cell death. Of note, the LDH assay indicated a slightly wider range of effective concentrations of the compounds, but the cytoprotective activity of the inhibitors in general showed a similar tendency (Figures 3, 4). The results of these analyses allowed for selection of the most effective concentrations of the UPR inhibitors for further studies (0.4 μM AMG44 for pre-treatment, 0.8 μM AMG44 for post-treatment, 1.6 μM JNK V for pre-treatment, and 0.8 μM JNK V for post-treatment, respectively).

### 3.3 Pre-treatment with UPR inhibitors partially preserves cell morphology

**FIGURE 5:**
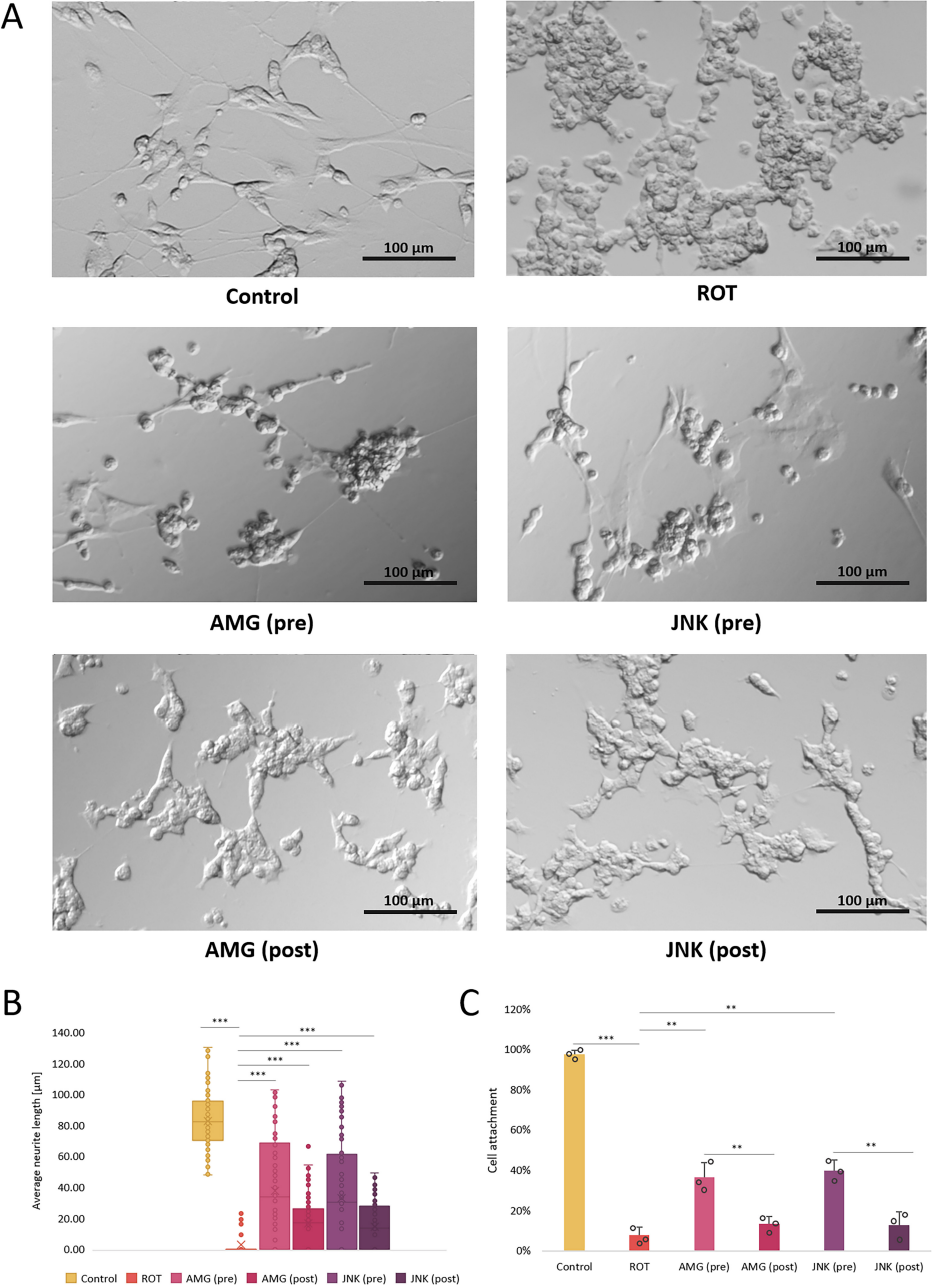
The morphological changes in differentiated SH-SY5Y cells incubated with rotenone (ROT) at 12.5 μM for 48 h and Unfolded Protein Response (UPR) inhibitors at their effective concentrations for 1 h; presented are representative pictures **(A)**, quantitative assessment of the average neurite length **(B)** and cell attachment count **(C)**. Control, untreated cells; ROT, cells treated with rotenone (48 h); AMG, cells treated with ROT (48 h) and AMG44 PERK inhibitor (1 h); JNK, cells treated with ROT (48 h) and JNK V inhibitor (1 h); pre, pre-treatment with the inhibitor; post, post-treatment with the inhibitor. Scale bar = 100 μm. Data were analyzed by the Kruskal-Wallis test with *post-hoc* Dunn’s test **(B)** or two-way ANOVA with Bonferroni correction **(C)**. Values are presented as median and interquartile range **(B)** or mean ± SD **(C)** (*n* = 3), **p < 0.01, ***p < 0.001 for all groups.

Differentiated SH-SY5Y cells were characterized by neuronal morphology as observed by elongated shape and multiple neurites. Treatment with ROT at 12.5 μM resulted in cell shrinkage, significant detachment and loss of neurites, indicative of exacerbated cell death. Pre-treatment with the inhibitors of both UPR signaling pathways, PERK- and IRE1-dependent, resulted in visible improvement of cell morphology as evidenced by a significantly higher proportion of attached cells with preserved neuronal projections. Post-treatment with the compounds slightly reversed ROT-induced damage, as there were present more attached cells with short neurites, but to a lesser extent than pre-treatment (Figure 5). Therefore, treatment with both AMG44 and JNK V partially protected differentiated SH-SY5Y cells against ROT-induced toxicity and morphological alterations.

### 3.4 PERK inhibition reduces caspase-3 activity

**FIGURE 6:**
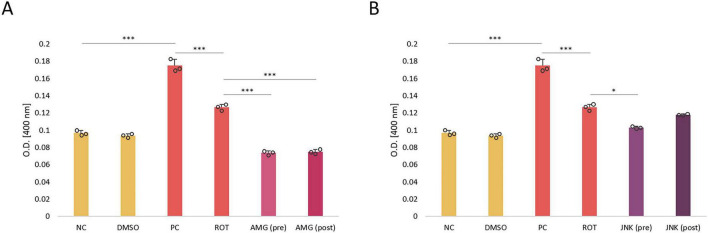
The level of caspase-3 activity in differentiated SH-SY5Y cells incubated with rotenone (ROT) at 12.5 μM for 48 h and UPR inhibitory compounds AMG44 **(A)** and JNK V **(B)** at their effective concentrations for 1 h. The caspase-3 level was evaluated by the respective colorimetric assay. Data were analyzed by the two-way ANOVA with Bonferroni correction. Values are presented as mean ± SD (*n* = 3), *p < 0.05, ***p < 0.001 for all groups. NC, Negative Control, untreated cells; DMSO, Solvent Control, cells treated with 0.1% dimethyl sulfoxide; PC, Positive Control, cells treated with 10 μM staurosporine (24 h); ROT, Cells Treated with Rotenone (48 h); AMG, cells treated with ROT (48 h) and AMG44 inhibitor (1 h); JNK, Cells Treated with ROT (48 h) and JNK V inhibitor (1 h); (pre), pre-treatment with the inhibitor; (post), post-treatment with the inhibitor.

The induction of apoptosis in differentiated SH-SY5Y cells incubated with ROT and UPR inhibitors was estimated based on the caspase-3 activity. Treatment of cells with ROT at 12.5 μM significantly increased caspase-3 activity, indicative of induction of apoptosis by the neurotoxin. Cells treated with PERK inhibitor AMG44, either before or after ROT-induced injury, exhibited significantly decreased caspase-3 activity, lower than that of negative control. On the contrary, pre-treatment with JNK V had little significant effect, whereas post-treatment had no significant effect on caspase-3 processing (Figure 6). Thus, it seemed that the neuroprotective effect of JNK V inhibitor did not depend entirely on caspase-3 processing.

### 3.5 UPR inhibitors target specific types of ROT-induced cell death

**FIGURE 7:**
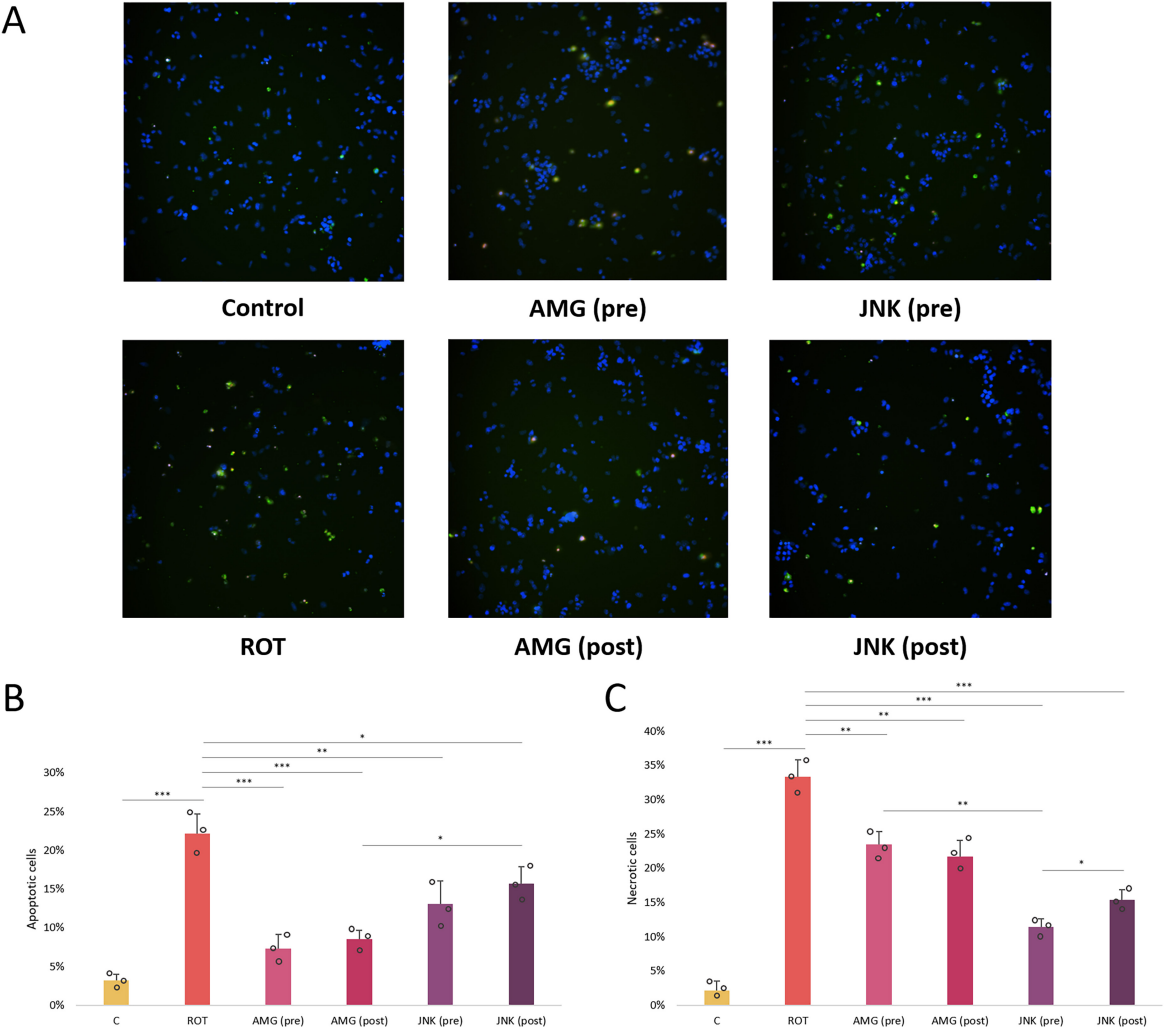
Microscopic images **(A)** and quantitative analysis **(B,C)** of the FITC-Annexin V (AV)/propidium iodide (PI) fluorescent staining of differentiated SH-SY5Y cells exposed to rotenone (ROT) at 12.5 μM for 48 h and UPR inhibitors at their effective concentrations for 1 h. The apoptotic AV-positive cells are characterized by green fluorescent signal, whereas the PI-positive dead cells present red fluorescent signal. The nuclei were counterstained with Hoechst (blue). The fluorescence was detected by the Nikon imaging system, and the intensity of FITC-AV/PI fluorescent signal was calculated. Data were analyzed by the two-way ANOVA with Bonferroni correction. Values are presented as mean ± SD (*n* = 3), *p < 0.05, **p < 0.01, ***p < 0.001 for all groups. Control, untreated cells; ROT, cells treated with rotenone (48 h); AMG, cells treated with ROT (48 h) and AMG44 inhibitor (1 h); JNK, cells treated with ROT (48 h) and JNK V inhibitor (1 h); (pre), pre-treatment with the inhibitor; (post), post-treatment with the inhibitor.

While our caspase-3-based apoptosis assessment showed that ROT triggered apoptosis in differentiated SH-SY5Y cells, previous experiments had shown that ROT-induced cell death was not limited to apoptosis, as it might also induce necrosis, especially at higher concentrations (Hartley et al., 1994; Hong et al., 2014). We therefore performed additional AV/PI fluorescent staining in differentiated SH-SY5Y cells treated with ROT and UPR inhibitors to distinguish between the effect of the compounds on specific types of cell death induced by ROT. Exposure of cells to 12.5 μM ROT for 48 h resulted in significant increase in AV+/PI+ and AV-/PI+ cells, indicative of late apoptosis and necrosis triggered by the neurotoxin. Both pre- and post-treatment with AMG44 PERK inhibitor significantly decreased the ratio of AV+ apoptotic cells, which corresponded with caspase-3 test results, and it also reduced the number of necrotic cells to a lesser degree. Interestingly, treatment with JNK V inhibitor, either before or after ROT-induced injury, significantly diminished the level of PI+ late apoptotic and necrotic cells (Figure 7). These results suggested that while the protective effect of PERK inhibition was mainly due to decline in apoptosis, JNK inhibition primarily exerted neuroprotection by reducing necrosis.

### 3.6 Inhibition of UPR pathways decreases the level of reactive oxygen species

**FIGURE 8:**
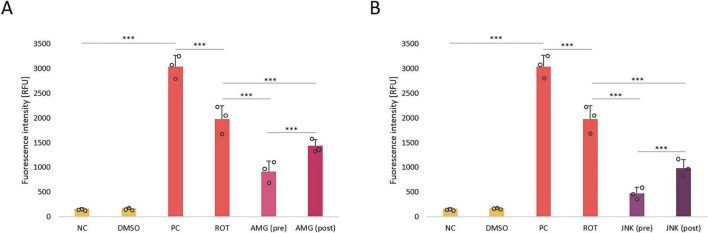
The analysis of reactive oxygen species (ROS) production in differentiated SH-SY5Y cells treated with rotenone (ROT) at 12.5 μM for 48 h and incubated with PERK inhibitor AMG44 **(A)** and JNK inhibitor JNK V **(B)** for 1 h at the effective concentrations. The ROS level was evaluated by the respective fluorometric assay. Data were analyzed by the two-way ANOVA with Bonferroni correction. Values are expressed as mean ± SD (*n* = 3), ***p < 0.001 for all groups. NC, Negative Control, untreated cells; DMSO, Solvent Control, cells treated with 0.1% dimethyl sulfoxide; PC, Positive Control, cells treated with 50 μM antimycin A (24 h); ROT, cells treated with rotenone (48 h); AMG, cells treated with ROT (48 h) and AMG44 inhibitor (1 h); JNK, cells treated with ROT (48 h) and JNK V inhibitor (1 h); (pre), pre-treatment with the inhibitor; (post), post-treatment with the inhibitor.

In the next step of our study, we aimed to investigate the effect of inhibition of PERK/CHOP and IRE1/JNK pathways on ROS generation in ROT-treated differentiated SH-SY5Y cells. As expected, incubation with 12.5 μM ROT, which is a mitochondrial neurotoxin, resulted in a significant induction of ROS in treated cells. Treatment with UPR inhibitors AMG44 and JNK V significantly reduced the amount of ROT-induced ROS, of which pre-treatments decreased the ROS levels more effectively than post-treatments, and JNK inhibitor resulted in a greater decrease in ROS generation than PERK inhibitor (Figure 8). This suggested that inhibition of PERK- and IRE1-dependent UPR pathways was protective against ROT-induced mitochondrial damage.

### 3.7 Inhibition of UPR pathways affects the expression of ER stress-related genes

**FIGURE 9:**
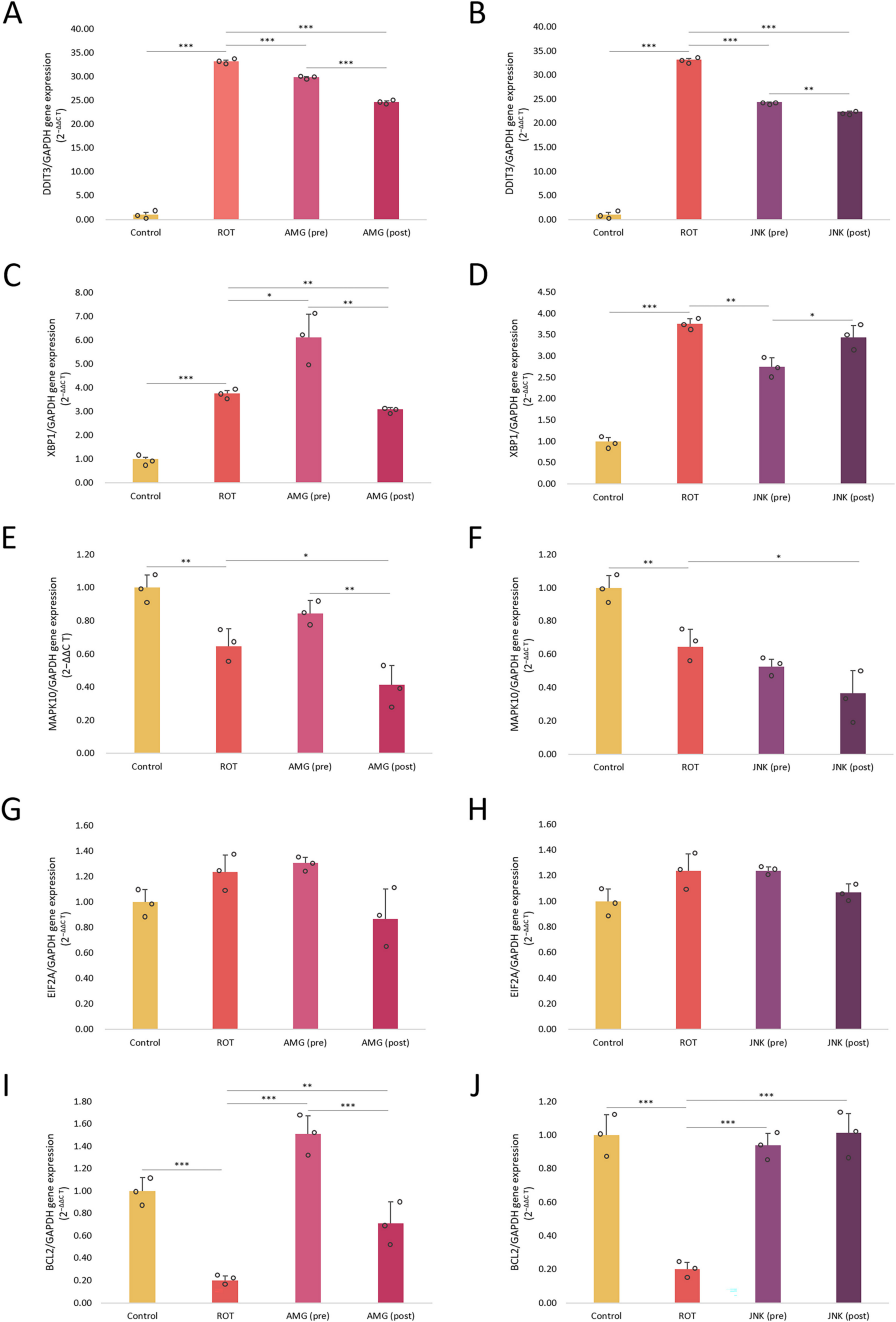
The mRNA expression levels of endoplasmic reticulum (ER) stress- and apoptosis-related genes *DDIT3* **(A,B)**, *XBP1* **(C,D)**, *MAPK10* **(E,F)**, *EIF2A* **(G,H)**, and *BCL2* **(I,J)** in differentiated SH-SY5Y cells exposed to rotenone (ROT) at 12.5 μM for 48 h and AMG44 **(A,C,E,G,I)** or JNK V **(B,D,F,H,J)** inhibitors at their effective concentrations for 1 h. *GAPDH* was used as a reference gene. Data were analyzed by the two-way ANOVA with Bonferroni correction. Values are presented as mean ± SD (*n* = 3), *p < 0.05, **p < 0.01, ***p < 0.001 for all groups. Control, untreated cells; ROT, cells treated with rotenone (48 h); AMG, cells treated with ROT (48 h) and AMG44 inhibitor (1 h); JNK, cells treated with ROT (48 h) and JNK V inhibitor (1 h); (pre), pre-treatment with the inhibitor; (post), post-treatment with the inhibitor.

Once we knew that inhibition of UPR pathways affected the viability, morphology, caspase-3 processing and ROS level in differentiated SH-SY5Y cells exposed to ROT, we wanted to evaluate the mechanism behind these changes. For this purpose, we examined the effect of PERK and JNK inhibitors on the expression of genes related to ER stress and apoptosis in ROT-treated cells. Incubation with ROT for 48 h induced a significant increase in *DDIT3* (∼30-fold) and total *XBP1* (∼4-fold) mRNA levels, and at the same time significantly decreased the expression levels of *MAPK10* and *BCL2* mRNA. Both pre- and post-treatments with UPR inhibitors significantly downregulated *DDIT3* gene, encoding the pro-apoptotic CHOP protein, and for both compounds post-treatments decreased the *DDIT3* expression to a higher extent than pre-treatments. Interestingly, pre-treatment with AMG44 slightly upregulated the *XBP1* gene related to the IRE1 pathway, while 1 h post-treatment with AMG44 and 1 h pre-treatment with JNK V significantly reduced the expression of this gene. This suggested that IRE1/XBP1 branch inhibition could be a long-term event associated with JNK inhibition and an early event triggered by PERK inhibition. As regards *MAPK10* gene, encoding JNK3 isoform, its expression was significantly reduced upon 1 h post-treatment with UPR inhibitors. Inhibition of PERK affected the *MAPK10* expression in a similar manner to that of *XBP1*, which indicated that inhibition of PERK in the early phases also inhibited IRE1-dependent signaling, whereas later on it switched UPR toward IRE1/XBP1/JNK branch activation. The expression of *EIF2A* gene remained stable and unaffected by either ROT or UPR inhibitors exposure. The anti-apoptotic *BCL2* gene was significantly downregulated by ROT, and upregulated upon pre- or post-treatment with UPR inhibitors, of which JNK V inhibitor restored the *BCL2* expression to the basal level as observed in control (Figure 9).

### 3.8 UPR inhibitors modulate the expression of ER stress-associated proteins

**FIGURE 10:**
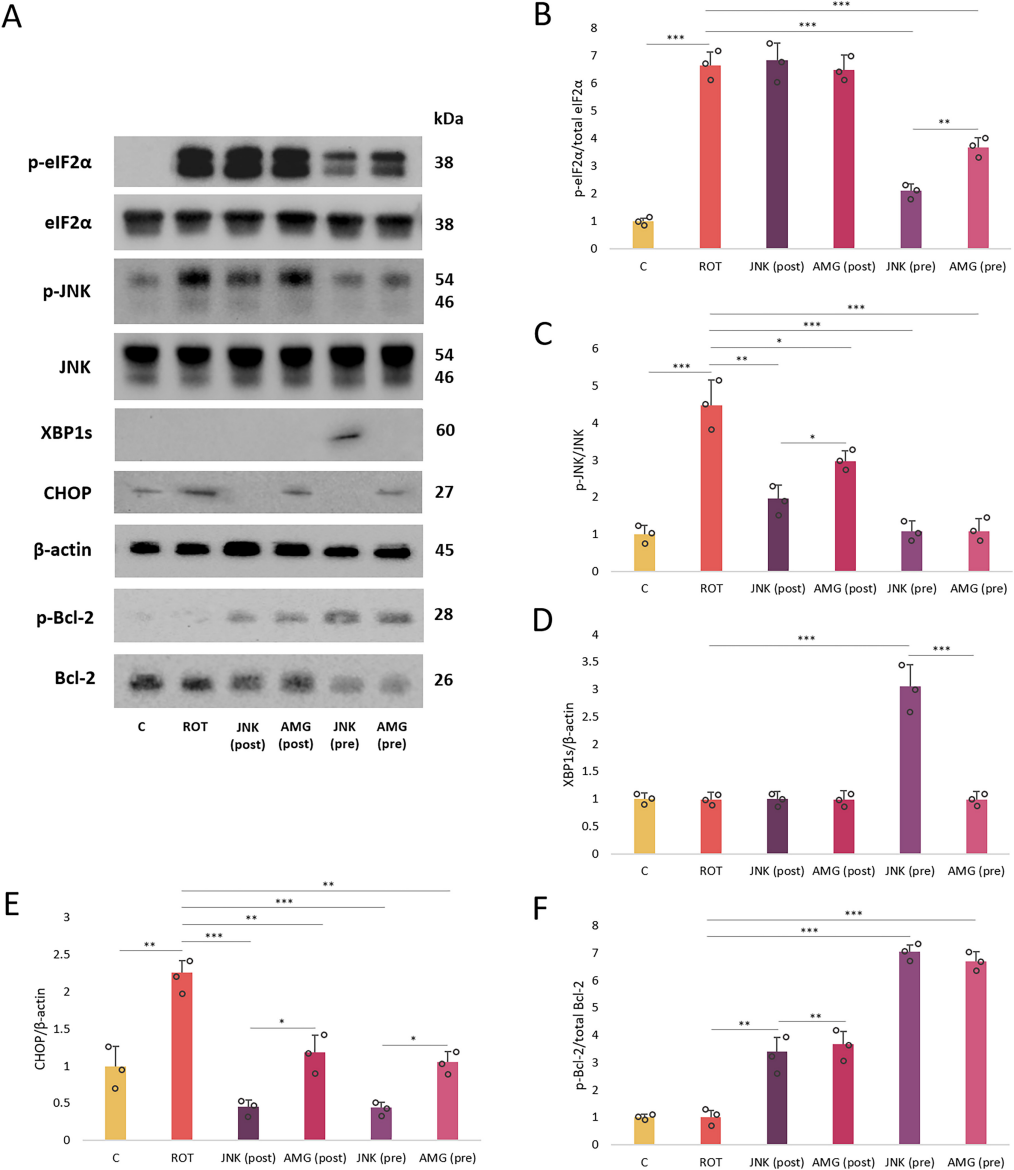
The Western blot images **(A)** and normalized protein expression levels of the endoplasmic reticulum (ER) stress- and apoptosis-related factors **(B–F)**: p-eIF2α **(B)**, p-JNK **(C)**, XBP1s **(D)**, CHOP **(E)**, and p-Bcl-2 **(F)** in differentiated SH-SY5Y cells incubated with rotenone (ROT) at 12.5 μM for 48 h, and AMG44 and JNK V compounds at their effective concentrations for 1 h. β-actin was used as a loading control. Data were analyzed by the two-way ANOVA with Bonferroni correction. Values are presented as mean ± SD (*n* = 3), *p < 0.05, **p < 0.01, ***p < 0.001 for all groups. Control, untreated cells; ROT, cells treated with rotenone (48 h); AMG, cells treated with ROT (48 h) and AMG44 inhibitor (1 h); JNK, cells treated with ROT (48 h) and JNK V inhibitor (1 h); (pre), pre-treatment with the inhibitor; (post), post-treatment with the inhibitor.

Immunoblotting was applied to further evaluate the mechanism behind neuroprotection provided by UPR inhibitors in ROT-based PD cellular model at the protein level. Exposure of differentiated SH-SY5Y cels to ROT for 48 h significantly upregulated the p-eIF2α, p-JNK, and CHOP levels, which indicated that the neurotoxin treatment triggered ER stress conditions and apoptosis. Pre-treatment with UPR inhibitors significantly reduced the phosphorylation of eIF2α and JNK, while post-treatment with the compounds did not affect p-eIF2α level but managed to decrease the level of p-JNK. Of note, the expression of cytoprotective XBP1s factor associated with the IRE1 pathway was induced by JNK V inhibitor pre-treatment only. Furthermore, treatment with UPR inhibitors significantly attenuated the expression of pro-apoptotic CHOP factor, both when applied before or after ROT-induced injury. While treatment with AMG44 downregulated CHOP approximately to the level of control, JNK V treatment completely abolished the expression of this protein. Lastly, treatment with the UPR inhibitory compounds upon ROT exposure induced the phosphorylation of Bcl-2, the major anti-apoptotic protein, and in this case pre-treatments were more effective than post-treatments (Figure 10).

## 4 Discussion

This study demonstrates that pharmacological inhibition of both PERK/CHOP and IRE1/JNK branches of the UPR protects differentiated SH-SY5Y cells from rotenone-induced toxicity. Treatment of ROT-injured cells with the selective UPR inhibitors improves cell viability and morphology, decreases ROS production, and limits both apoptotic and necrotic cell death. PERK inhibition mainly suppresses caspase-3-dependent apoptosis, while JNK inhibition more effectively reduces necrosis. These observations underscore the neuroprotective potential of modulating ER stress pathways in PD.

Mechanistically, PERK and JNK inhibitors differentially modulate key ER stress markers and pro-apoptotic factors, including eIF2α, CHOP, XBP1, JNK, and Bcl-2. First, the anti-apoptotic effect of PERK inhibition depends primarily on decreasing caspase-3 activity, whereas JNK inhibition provides neuroprotection mainly by reducing ROT-induced necrotic cell death. Second, both compounds affect *XBP1* mRNA expression. Last, JNK inhibition induces long-term expression of the XBP1s protein, whereas PERK inhibition does not. Inhibition of JNK, when compared to PERK, more effectively decreases the ROS level, the expression of *DDIT3* and *MAPK10* mRNA expression, and the levels of p-eIF2α, p-JNK, and CHOP proteins. Strikingly, under ROT-induced ER stress, inhibition of either PERK/CHOP or IRE1/JNK axis leads to downregulation of the other, demonstrating reciprocal cross-talk between the two UPR pathways and highlighting the pleiotropic actions of these inhibitors in alleviating ER stress and UPR-mediated proapoptotic signaling.

Here, we demonstrate that JNK inhibition better protects against ROT-induced oxidative stress than PERK inhibition in differentiated SH-SY5Y cells. This may be due to JNK’s central role in controlling mitochondrial ROS generation in a feedback loop (Heslop et al., 2020). Thus, JNK inhibition directly and indirectly lowers ROS by shifting the IRE1-dependent pathway to the pro-survival, antioxidant XBP1s axis. In contrast, PERK activates the antioxidant Nrf2 factor (Saptarshi et al., 2022), which is suppressed when PERK is inhibited. This suggests that targeting CHOP (a downstream of PERK) rather than PERK itself may be a better option, although no pharmacological CHOP inhibitors currently exist.

In our previous study, we evaluated AMG44 and JNK V inhibitors in another neurotoxic cellular model of PD—differentiated SH-SY5Y cells exposed to 6-hydroxydopamine (6-OHDA). In the 6-OHDA-based model, JNK inhibition more effectively increases cell viability, decreases genotoxicity, and modulates ER stress marker expression, whereas PERK inhibition reduces caspase-3 levels more strongly. By contrast, in the ROT model, both PERK and JNK inhibitors are similarly effective at restoring cell viability. This discrepancy could stem from the distinct mechanisms of action of each toxin: rotenone mainly inhibits mitochondrial complex I, whereas 6-OHDA induces more extensive oxidative damage (Giordano et al., 2012; Okyere et al., 2021; Simões et al., 2022). Therefore, the acute oxidative damage caused by 6-OHDA may exceed the protective capacity of PERK inhibition alone. In the ROT model, modulation of PERK signaling may be protective because PERK affects mitochondrial complex I function through multiple mechanisms (Balsa et al., 2019; Zhang et al., 2021). JNK regulates the activity of other mitochondrial complexes and ROS formation (Dougherty et al., 2004; Perez-Gomez et al., 2020), with the JNK substrate Bcl-2 acting as a mitochondrial ROS sensor through complex IV modulation (Chong et al., 2014; Kotrasová et al., 2021). Other known JNK downstream effectors—Bim, p53, or p73—also influence complex IV activity, possibly providing broader protection against strong oxidizing agents like 6-OHDA (Rufini et al., 2012; Saleem et al., 2015; Wali et al., 2018). In both models, PERK inhibition potently reduces caspase-3 levels compared to JNK inhibition. This supports the notion that JNK3 neuroprotection in dopaminergic neurodegeneration could involve caspase-3-independent pathways and other mechanisms of ROT-induced cell death (Han et al., 2014). Indeed, FITC-AV/PI counterstaining in the present study shows that ROT induces both apoptosis, which PERK inhibition effectively abolishes, and necrosis, which JNK inhibition reduces more strongly. This aligns with previous findings that JNK can mediate both apoptosis and necrotic cell death in a TNF-dependent manner (Li et al., 2019; Ventura et al., 2004; Win et al., 2011). While the JNK inhibitor exerts neuroprotection in both models by blocking a key cell death-associated kinase, the actual role of PERK in dopaminergic neurodegeneration seems to be more complex and yet to be fully uncovered.

To date, the effects of AMG44 and JNK V inhibitors in the ROT-based PD model have not been investigated. Previously, PERK inhibition by GSK2606414 demonstrated strong neuroprotective effects in PD mice, but its clinical applicability is limited by pancreatic toxicity (Mercado et al., 2018). Hence, there is a need to develop a new generation of PERK inhibitors that do not exhibit such off-target events. For AMG44, preliminary studies suggest that it does not affect the pancreas function (Mohamed et al., 2020). Still, data on tissue distribution and blood-brain barrier (BBB) permeability of AMG44 are lacking, and these essential aspects need to be tested. Both AMG44 and JNK V inhibitors are highly selective, and JNK V targets the CNS-specific JNK3 isoform, which limits potential peripheral effects. JNK V was previously shown to cross the BBB and exhibit neuroprotection against ischemic stroke in a gerbil model (Carboni et al., 2008). A separate study found that JNK V inhibitory action is not limited to JNK and can affect other kinases, leading to off-target effects and unintended consequences (Kim et al., 2013). Hence, all UPR inhibitory compounds require careful *in vivo* evaluation before being implemented in clinical trials. Specifically, pharmacokinetics/pharmacodynamics (PK/PD) profile, efficacy, safety, target engagement and off-target activity of the drug, as well as disease heterogeneity, should be considered before initiating large clinical trials. Medicinal-chemistry optimization and targeted delivery strategies (e.g., prodrugs, receptor-mediated transport, focused ultrasound) could also help improve BBB penetration, and concrete tactics (dosing, PK/PD control, delivery choices, high isoform selectivity, kinome profiling, and early safety monitoring) could minimize off-target effects.

Our results align with previous studies that identified ER stress as a central player in ROT-induced neuronal death. For instance, depletion of *MAPK10* encoding JNK3 protected dopaminergic neurons against paraquat- and ROT-induced cell death and rescued motor deficits *in vivo* (Choi et al., 2010). A separate study has also confirmed an essential role of JNK3 in ROT-induced dopaminergic degeneration, and the microtubule stabilizing agent paclitaxel effectively attenuated JNK activation (Choi et al., 2015). A specific JNK inhibitor SP600125 was effective against ROT-induced damage in dopaminergic neurons (Choi et al., 2015) and SH-SY5Y cells (Ma et al., 2018). The other compound, an eIF2α activator salubrinal, ameliorated ROT-induced ER stress and apoptosis in SH-SY5Y (Wu et al., 2014) and Neuro2a cells (Gupta et al., 2019). In ROT-treated rats, among various UPR modulators (YM08, 4 μ8C, AEBSF, or ursolic acid), salubrinal showed the most potent neuroprotective effect, as evidenced by the restoration of biochemical alterations, neuronal viability, and morphology (Gupta et al., 2021). Genetic downregulation of eIF2α expression also protected SK-N-MC cells against ROT-induced death (Chen et al., 2008). Collectively, these data suggest that fine-tuning of eIF2α activity could be a key to providing neuroprotection during PD. In this study, AMG44 and JNK V inhibitors partially decrease eIF2α phosphorylation, a desirable effect that increases cell survival upon ROT-induced damage.

Intriguingly, several antidiabetic drugs were effective in preclinical ROT-based PD models by modulating ER stress. Metformin, a type II diabetes mellitus (DM) drug, protected mice from ROT-induced PD. It reduced ER stress and the neuroinflammatory response by downregulating GRP78, XBP1, ATF4, ATF6, CHOP, and IL-1β and TNF-α cytokines (Wang et al., 2020). In ROT-administered rats, repaglinide restored striatum morphology and motor performance by decreasing GRP78, CHOP, ATF6, and caspase-3 levels; this reduced apoptosis, oxidative stress, and inflammation. In the same model, omarigliptin also downregulated GRP78, CHOP, and caspase-12, leading to antioxidant, anti-inflammatory, and anti-apoptotic effects, lower α-synuclein aggregation, and improved motor function. As the drug crosses the BBB, it may serve as a potential PD treatment (Michel et al., 2022). Empagliflozin reduced GRP78/PERK/eIF2α/CHOP activity, miR-211-5p level, oxidative stress and gliosis, and improved autophagy and motor function in ROT-injected rats. These findings are especially promising for PD patients with coexisting DM. Among other FDA-approved drugs, hydroxocobalamin (vitamin B12 analog) lessened ROT damage in SH-SY5Y by lowering PERK-mediated eIF2α phosphorylation (Jeon et al., 2022). In PC12 cells, rifampicin protected against ROT by inducing the GRP78/PERK/eIF2α/ATF4 axis (Jing et al., 2014), while nicardipine protected SH-SY5Y cells by inhibiting JNK, p38 MAPK, and caspases (Park and Kim, 2013). Fluoxetine reduced apoptosis and catalepsy and improved movement by decreasing GRP78, XBP1, CHOP, and caspase-3 levels in ROT-treated rats (Peng et al., 2018), suggesting therapeutic potential for PD patients with depression.

Several natural compounds, including polyphenols and flavonoids, have also been tested in ROT models of PD. These compounds exhibit pleiotropic effects by reducing ER stress, oxidative stress, and apoptosis across various PD models. For example, quercetin lowered oxidative and ER stress levels in ROT-induced PD rats. In ROT-treated mice, hydroxysafflor yellow A enhanced the formation of the autophagosome and promoted α-syn clearance by increasing, inter alia, JNK1 and Bcl-2 phosphorylation (Han et al., 2018). Phloretin in the same model improved anxiety-like behavior, locomotor activity, and neural survival by upregulating the antioxidant Nrf2, a downstream target of PERK (Shirgadwar et al., 2023). Geraniol reduced the levels of PERK, eIF2, IRE1α, ATF6, ATF4, and CHOP in ROT-treated SK-N-SH cells, thereby diminishing oxidative stress, improving mitochondrial function, and enhancing autophagy flux (Rekha and Inmozhi Sivakamasundari, 2018). Finally, in SH-SY5Y cells exposed to ROT, a number of compounds exerted neuroprotective properties: proanthocyanidins by inhibiting p38, JNK, and ERK signaling (Ma et al., 2018), naringin by suppressing p38, JNK, Bcl-2, and Bax (Kim et al., 2009), and fisetin – by downregulating Bax and caspase-3, and upregulating, among others, Bcl-2, p38 and JNK (Rajendran and Ramachandran, 2019). Interestingly, overexpression of growth hormone provided resistance to ROT-induced toxicity in SH-SY5Y and SK-N-AS neuroblastoma cell lines by modulating the expression of CHOP, PERK, XBP1, and ATF6 (Rencüzoğullari et al., 2023). What is more, in SH-SY5Y cells exposed to ROT, a secretome obtained from neural-induced human adipose tissue-derived stem cells reduced the levels of p-PERK, p-IRE1α, p-SAPK, ATF4, and CHOP, thereby restoring mitophagy, mitochondrial fusion, and tethering at mitochondria-associated membranes (Ramalingam et al., 2023). In the same cellular model, inhibition of miR-384-5p significantly suppressed ROT-evoked ER stress and toxicity by downregulating the expression of UPR proteins GRP78, ATF4, IRE1α, XBP1, and CHOP (Jiang et al., 2016).

While this study provides novel insights into the involvement of UPR pathways in ROT-induced neurotoxicity, several limitations should be acknowledged. First, all experiments were performed in vitro using a differentiated, immortalized tumor-derived cell line. Although widely accepted as a PD model, it cannot fully mimic the complexity of human neuronal networks. The limited sample size and potential differentiation heterogeneity may also introduce experimental variability. Second, our findings rely on pharmacological modulation of the UPR. Neurotoxicity was induced by acute exposure to a chemical toxin, which may not fully represent the chronic, multifactorial nature of PD (Castillo-Rangel et al., 2023; Çınar et al., 2022; Grotemeyer et al., 2022; Tansey et al., 2022). While the inhibitors’ specificity was verified based on prior reports, potential off-target effects cannot be entirely excluded. Third, we investigated single time points and did not assess the long-term effects of treatment. Finally, our study lacked genetic validation of the targeted pathways (e.g., siRNA or CRISPR knockdown). Future research should address these limitations by testing PERK and JNK inhibition in more representative systems (e.g., iPSC-derived dopaminergic neurons, 3D organoid cultures). Incorporating glial components or co-culture systems may better reflect the PD microenvironment, especially regarding neuroinflammation. Moreover, *in vivo* studies are needed to assess pharmacokinetics, blood–brain barrier penetration and systemic tolerability. The α-synuclein-based rodent models could serve as a valuable platform for systemic evaluation of the UPR inhibitors. All mentioned approaches could strengthen the translational potential of the present findings.

Taken together, our data suggest that pharmacological inhibition of both the PERK/CHOP and IRE1/JNK pathways of the UPR protects differentiated SH-SY5Y cells against ROT-induced toxicity. First, upon ROT treatment, AMG44 and JNK V inhibitory compounds increase cell viability, as evaluated by the two independent assays. Second, UPR inhibitors improve neural morphology during ROT exposure, as evidenced by decreased cell detachment and preserved neurites. Third, the evaluated PERK inhibitor strongly decreases caspase-3 levels and thus limits apoptosis, whilst the JNK inhibitor more potently inhibits ROT-induced necrotic cell death. Last, both drugs reduce ROT-dependent ROS production and oxidative damage in differentiated SH-SY5Y cells. Mechanistically, both compounds exert neuroprotection by modulating the expression of specific pro-apoptotic ER stress-related factors, and inhibition of one UPR pathway affects the other, indicative of crosstalk between PERK/CHOP and IRE/JNK signaling. The biological activity of these two compounds should be further evaluated in more advanced cellular models and *in vivo*. Especially, the effectiveness of post-treatment with UPR inhibitors sheds light on the potential future use of these compounds in clinical practice.

## Data Availability

The raw data supporting the conclusions of this article will be made available by the author, without undue reservation.
